# Concatenation of 14-3-3 with partner phosphoproteins as a tool to study their interaction

**DOI:** 10.1038/s41598-019-50941-3

**Published:** 2019-10-18

**Authors:** Kristina V. Tugaeva, Daria I. Kalacheva, Richard B. Cooley, Sergei V. Strelkov, Nikolai N. Sluchanko

**Affiliations:** 1 A.N. Bach Institute of Biochemistry, Federal Research Center of Biotechnology of the Russian Academy of Sciences, 119071 Moscow, Russia; 2 Department of Biochemistry, School of Biology, M.V. Lomonosov Moscow State University, 119991 Moscow, Russia; 3 Department of Biochemistry and Biophysics, Oregon State University, Corvallis, OR 97331 USA; 4 Laboratory for Biocrystallography, Department of Pharmaceutical and Pharmacological Sciences, KU Leuven, 3000 Leuven, Belgium; 5 Department of Biophysics, School of Biology, M.V. Lomonosov Moscow State University, 119992 Moscow, Russia

**Keywords:** Proteins, Structural biology, Intrinsically disordered proteins, Molecular biophysics

## Abstract

Regulatory 14-3-3 proteins interact with a plethora of phosphorylated partner proteins, however 14-3-3 complexes feature intrinsically disordered regions and often a transient type of interactions making structural studies difficult. Here we engineer and examine a chimera of human 14-3-3 tethered to a nearly complete partner HSPB6 which is phosphorylated by protein kinase A (PKA). HSPB6 includes a long disordered N-terminal domain (NTD), a phosphorylation motif around Ser16, and a core α-crystallin domain (ACD) responsible for dimerisation. The chosen design enables an unstrained binding of pSer16 in each 1433 subunit and secures the correct 2:2 stoichiometry. Differential scanning calorimetry, limited proteolysis and small-angle X-ray scattering (SAXS) support the proper folding of both the 14-3-3 and ACD dimers within the chimera, and indicate that the chimera retains the overall architecture of the native complex of 14-3-3 and phosphorylated HSPB6 that has recently been resolved using crystallography. At the same time, the SAXS data highlight the weakness of the secondary interface between the ACD dimer and the C-terminal lobe of 14-3-3 observed in the crystal structure. Applied to other 14-3-3 complexes, the chimeric approach may help probe the stability and specificity of secondary interfaces for targeting them with small molecules in the future.

## Introduction

14-3-3 proteins are abundant cytosolic factors involved in the regulation of many physiological processes like apoptosis, cell division, ion channel functioning, signaling, cytoskeleton rearrangements. 14-3-3 proteins are expressed in all studied eukaryotes, typically being represented by several isoforms per organism^1^. In humans, there are seven 14-3-3 isoforms designated by Greek letters (β, ζ, τ, η, ε, σ, γ) and encoded by separate genes^2^. These 30-kDa polypeptides form stable homo- and heterodimers^3,4^ and are widely distributed in mammalian tissues, reaching especially high levels in the nervous system (≥1% of proteome^5^).

The dimeric form is considered the main functional unit of 14-3-3. It has a W-like shape and consists of stacks of antiparallel α-helices^6,7^. The subunits contact each other in an antiparallel fashion by forming the vast interface between N-terminal α1-α2 helices from one subunit and α3-α4 helices from the second subunit, including a range of hydrophobic and polar interactions, as well as several salt bridges^8,9^. The C-terminal α-helices create the walls of the assembly that end with the unstructured flexible tails. The role of the latter remains unclear, although they were suggested to improve the solubility of 14-3-3 and modulate interactions with partner proteins by restricting the access to the main binding sites on 14-3-3^6,10,11^.

Each 14-3-3 protomer harbors the so-called amphipathic groove (AG), where the primary binding of phosphotarget proteins takes place. 14-3-3 proteins were the first phosphoserine/threonine recognizing protein modules discovered^12^ and they continue to attract increasing attention as the number of interacting phosphopartners discovered grows^13^. Bioinformatics analysis reveals thousands of “client” proteins containing putative 14-3-3 binding motifs phosphorylatable by a range of protein kinases, thereby making 14-3-3 important nodes in protein-protein interaction (PPI) networks^14–16^. Phosphorylation serves as a selective filter, promoting client binding to 14-3-3 when client is phosphorylated, and preventing binding when the same client is not phosphorylated. Phosphopeptide binding in the AGs of 14-3-3 preferentially occurs when the phosphoresidue embedded consensus motifs reside within intrinsically disordered regions (IDRs) of protein partners^17^ that contain sufficient flexibility required for binding to a rather rigid 14-3-3 dimer^18–22^. Motif I (R[S/F/Y/W]XpSXP), motif II (RX[S/Y/F/W/T/Q/A/D]Xp(S/T)X[P/L/M]) and motif III (pSX_1–2_–COOH), where pS/T is phosphorylated serine or threonine, have been characterised^23,24^, although targets deviating from these canonical motifs have also been reported^25,26^. In line with the disorder-to-order transition (folding) upon binding principles^20,22^, 14-3-3 proteins are thought to significantly restrict the conformational space sampled by the phosphopeptides within the complex, thereby being able to affect partner protein conformation and activity^27^. Possessing two AGs, the 14-3-3 dimer displays a maximum of two valences with respect to the binding phosphopeptides – bidentate binding of doubly phosphorylated targets to 14-3-3 usually is much more affine^24,28^. These principles of multisite binding are commonly employed in case of other proteins containing IDRs^29–31^. For many binary complexes involving 14-3-3, the affinity is expected to be rather low due to a transient character of interactions in the regulatory circuits. This suggests that fusion of transiently interacting partners may be a promising approach in structural biology of 14-3-3 complexes as it ensures the formation of a stable complex with correct stoichiometry.

The prevalence of IDRs poses challenges for crystallisation of 14-3-3/phosphotarget complexes due to the high flexibility and heterogeneity, whereas their relatively big sizes (>60 kDa) limit the utilization of nuclear magnetic resonance (NMR) spectroscopy. Unfortunately, those very sizes most of the time are too small for a successful application of cryo-electron microscopy (Cryo-EM). This at least partly explains the remarkable disproportion between the large number of discovered 14-3-3 interactors and a handful of the 14-3-3 complexes characterised with atomic precision (highlighted in^13^). The first crystal structure was reported in 2001 for the complex of 14-3-3ζ dimer with two aralkylamine N-acetyltransferase molecules^32^, whereas the second structure, with the dimer of the small heat shock protein HSPB6^33^, appeared only sixteen years later. During that time, the number of discovered 14-3-3 partners increased manifold.

**Figure 1:**
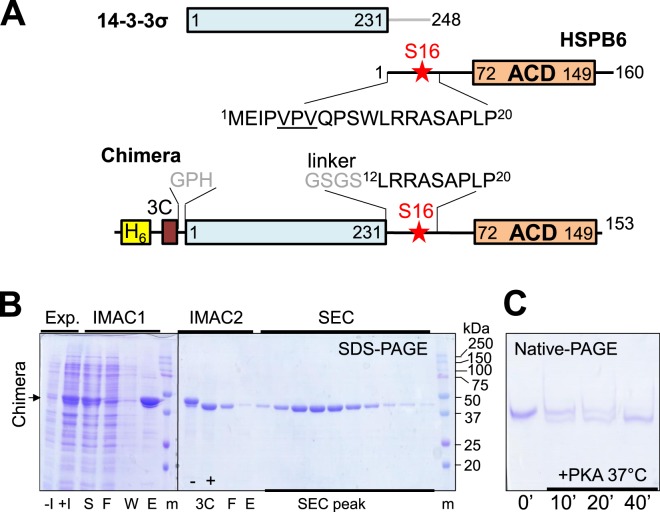
Design and purification of the 14-3-3-pB6 chimera. (**A**) Schematics showing the primary structure of both individual proteins and the chimera. The N-terminal hexahistidine-tag cleavable by 3C protease is shown. (**B**) Expression (Exp.) and chromatographic (IMAC1, IMAC2, SEC) purification of the unphosphorylated chimera as analyzed by SDS-PAGE. Qualitatively similar results were obtained after co-expression with PKA, but with a lower yield (not shown). Non-induced (−I), induced (+I), soluble (S), flowthrough (F), wash (W), eluted (E) fractions, and fractions collected during SEC (SEC peak) are indicated below the gel. Protein markers (m) with the corresponding masses (in kDa) are also indicated. Position of the chimera band is shown by an arrow to the left. Note the shift after the 3C proteolysis (3C: “−”, “+”). (**C**) Kinetics of *in vitro* phosphorylation of the purified chimera by PKA analyzed by native-PAGE. Note the downward shift corresponding to phosphate group incorporation.

HSPB6 belongs to the small heat shock protein family and consists of two major domains, namely the N-terminal domain (NTD) which is intrinsically disordered to a large part, and the α-crystallin domain (ACD) (Fig. 1A) which features a compact immunoglobulin-like fold and is responsible for dimerisation^34^. Upon HSPB6 phosphorylation at Ser16 in each of the chains, a 2:2 complex with 14-3-3 is formed^35–37^, which is thought to regulate smooth muscle and fibroblast relaxation via cAMP and cGMP signalling activation^38^ and, therefore, this interaction was considered a drug target in asthma^39^, erectile dysfunction^40^, fibrosis and excessive scarring^41^.

The crystal structure of the 14-3-3σ/pHSPB6 complex has revealed that the ACD dimer is asymmetrically docked onto the C-terminal lobe of one 14-3-3 subunit^33^. Besides a contact between the ACD and the 14-3-3 monomer, the observed conformation appears to be driven by the specific interactions of the NTD, a significant portion of which (first 38 out of 70 residues) becomes ordered upon the crystal formation. Specifically, residues 2–10 of the NTD patch the so-called β4/β8 groove of HSPB6 ACD, whereby the hydrophobic residues of the ^5^Val-Pro-Val^7^ motif insert into the hydrophobic core of the ACD. Such patching is generally known to play a key role in the oligomer assembly of the small heat shock proteins^34^. Next, the motif (residues 12–20) carrying phosphoserine-16 binds in the AG of 14-3-3. Finally, the so-called shared groove (β3/β3 groove) of the ACD dimer is patched by residues 27–34. Such a multipoint interaction implies an intricate binding mechanism, but the hierarchy of binding steps remains unaddressed. In particular, it is unknown how tight and specific binding of the HSPB6 ACD dimer is to the C-lobe of 14-3-3 once the HSPB6 phosphopeptides capture the latter.

Other crystal structures reported recently have been those of yeast 14-3-3 protein Bmh1 complexed with neutral trehalase Nth1 at a 2:1 stoichiometry^42^ and of 14-3-3β with non-phosphorylated exotoxins ExoS/ExoT at either 2:1 or 2:2 stoichiometry^25^. These few structures revealed that the secondary interfaces may be important for maintaining the architecture of 14-3-3 complexes, although whether they are sufficiently affine and specific on their own without the phosphopeptide binding remains a question difficult to address. At the same time, those secondary interfaces only slightly overlap among the known 14-3-3 complexes and, therefore, are very attractive for designing new-generation selective small-molecule modulators of the 14-3-3 PPIs^13,33,43^.

To promote structural studies, the chimeric constructions where the 14-3-3 core is tethered to an interacting fragment of a protein partner have recently been proposed^44^. This approach has been successfully tested on several peptide-fused prototypes, where the attached small target peptide was phosphorylated at the desired position via co-expression with the appropriate protein kinase^45^ and bound at the 14-3-3 AGs in the correct stoichiometry. Straightforward expression, purification, and crystallisation of such chimeras demonstrated equivalence of the structural information compared with the traditional co-crystallisation of 14-3-3 with synthetic phosphopeptides. The chimeric approach conferred a set of advantages and was not principally limited to short peptides used so far^44^, making it potentially applicable to longer fragments, but this question remained open.

The current work deals with the first attempt to use the chimeric approach for structural studies of 14-3-3 complexes with a complete phosphopartner, with the ambition to use it for 14-3-3 complexes generally, in a high-throughput format, in the future.

## Results

### Design of the 14-3-3-pB6 chimera

The C-terminal intrinsically disordered peptide of 14-3-3 does not affect the phosphopeptide-binding principles and is often removed for structural studies^33,43,44,46^. The last residue 231 of the structured 14-3-3 core is located in the vicinity of its amphipathic groove (AG). Based on these observations, we have recently constructed a C-terminal fusion of the HSPB6 peptide LRRAS^16^APL to 14-3-3σ, wherein the Ser16 residue could be phosphorylated by PKA. This chimeric protein termed pCH1 was soluble and could be crystallised. The X-ray structure showed phosphopeptide—14-3-3 interactions equivalent to those of 14-3-3 in complex with the synthetic, unfused HSPB6 phosphopeptide^33,44^. Given the N-terminal location of the 14-3-3-binding motif in the HSPB6 primary structure (a common feature of many 14-3-3 partners) and the native 2:2 stoichiometry of the 14-3-3–pHSPB6 complex^33^, the peptidic chimera construct was extended to a major part of HSPB6 including the NTD starting with residue 12 as well as the core ACD, yielding a chimera phosphorylatable at Ser16 of the HSPB6 part which we named “14-3-3-pB6” (Fig. 1A).

The fusion was expressed in *E*. *coli* in a soluble form in the presence or the absence of PKA and could be readily purified following the approach^44^ developed for the peptidic chimera (Fig. 1B). The unphosphorylated version was produced in much higher yield and was efficiently phosphorylated by PKA *in vitro* (Fig. 1C). As a result, milligram amounts of the electrophoretically homogenous phosphoprotein could be obtained in three days including expression.

### Oligomerisation of the 14-3-3-pB6 chimera

**Figure 2:**
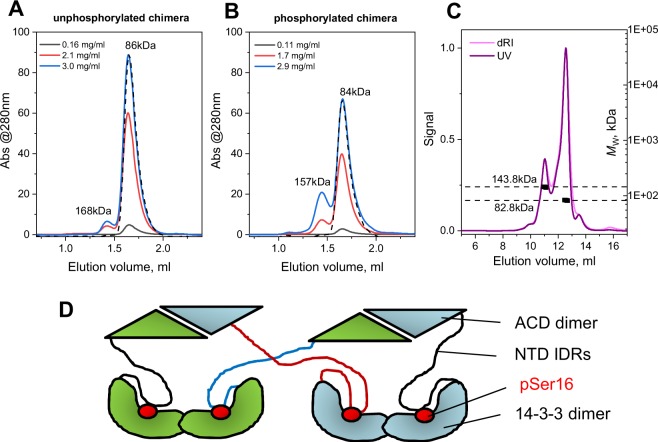
Oligomeric state of the 14-3-3-pB6 chimera. SEC profiles of either unphosphorylated (**A**) or PKA-phosphorylated (**B**) chimera loaded on a Superdex 200 Increase 5/150 column at different concentrations (indicated in mg/ml) and run at a 0.45 ml/min flow rate. *M*_W_ values of the peaks were determined from column calibration. Dashed lines show the lowest concentration profiles scaled to the main peak of the highest concentration profiles for clarity. (**C**) Absolute *M*_W_ values for the main two peaks of the phosphorylated chimera obtained by SEC-MALLS on a Superdex 200 Increase 10/300 column operated at a 0.5 ml/min flow rate. (**D**) Formation of chimera tetramers due to the domain swapping between two different chimera dimers. NTD IDRs – intrinsically disordered regions of the HSPB6 N-terminal domain.

To study the oligomeric state of the chimera, we employed size-exclusion chromatography (SEC) at different loaded protein concentrations of either unphosphorylated or phosphorylated version (Fig. 2A,B). Importantly, at low protein concentration both versions displayed on the elution profile a single symmetrical peak with the apparent *M*_W_ of ~85 kDa. The phosphorylated version had a slightly lower *M*_W_ value, which is likely because phosphorylation leads to the AG occupation by the HSPB6 phosphopeptide and, therefore, to protein compaction. At higher concentrations, a second peak with an apparent *M*_W_ of 157–168 kDa started to appear, again showing a slightly more compact particle size for the phosphorylated chimera, in which case also the amplitude was appreciably higher (Fig. 2). These data are in good agreement with the theoretical values for the dimer and tetramer (*M*_w_ = 83.4 kDa and 166.8 kDa, respectively). More accurate *M*_W_ estimates of the 14-3-3-pB6 chimera were obtained by SEC-MALLS: two peaks with *M*_w_ = 82.8 (76.1% mass fraction) and *M*_w_ = 143.8 kDa (23.9% mass fraction) were detected (Fig. 2C). The first peak with a negligible polydispersity (±1.05%) almost perfectly matched a dimer, while the second corresponded to a heterocomplex (Fig. 2D).

Natural 14-3-3 proteins form highly soluble and rather stable dimers maintained by extensive contacts involving N-terminal α-helices, and have short flexible C-terminal tails^6,8^. A C-terminal fusion partner would not likely affect dimerisation of the 14-3-3 core, however, the question remains whether the ACD part of the fusion is folded as in native HSPB6, whose oligomeric status is dictated by the ACD dimerisation^34,47^. Since the highest-order most stable oligomeric species is a dimer for both 14-3-3σ and the HSPB6 ACD, we presumed that the observed chimera tetramerisation occurs via domain swapping with the preservation of the 14-3-3 dimer and ACD dimer interfaces (Fig. 2D). Such swapping has been observed in some crystal structures of the peptidic 14-3-3 chimeras^44^ and is possible only if both the 14-3-3 dimer and ACD dimer are folded within the chimera and display their inherent propensity for dimerisation.

If ACD would be losing its dimerisation propensity, the highest possible distinct oligomer would always be dimer connected via 14-3-3 intersubunit contacts. These data suggest that the 14-3-3 and ACD parts are folded within the obtained chimera, prompting us to study this directly by differential scanning calorimetry (DSC) and limited proteolysis.

### Domain structure of the chimera studied by DSC and limited proteolysis

**Figure 3:**
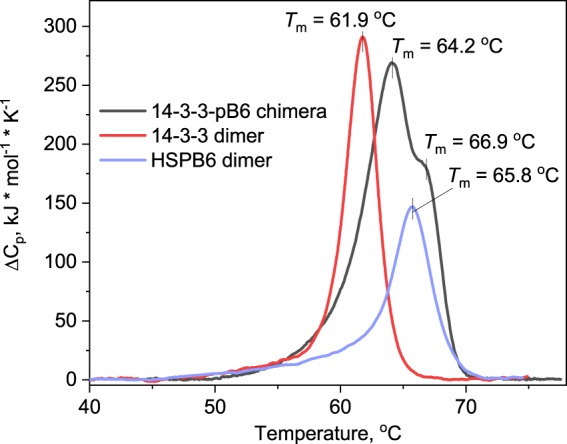
Analysis of the presence of folded domains within the 14-3-3-pB6 chimera by DSC. The samples containing either the 14-3-3 dimer, the HSPB6 dimer, or the 14-3-3-pB6 chimera were subjected to DSC at a constant heating rate of 1 °C/min. Thermal transition temperatures for the peaks (*T*_m_) are indicated as the positions of their maximum.

DSC was employed to study the thermal unfolding of the chimera compared to that of individual dimers of 14-3-3 and HSPB6 and to check for the presence of folded domains in its structure. In line with the previous reports^44,48,49^, the thermogram for the 14-3-3 dimer showed the cooperative transition with *T*_m_ = 61.9 °C, whereas HSPB6 revealed a transition with a higher *T*_m_ of 65.8 °C (Fig. 3). The chimera unfolded in at least two stages showing two distinct peaks on the thermogram characterised by *T*_m_ values of 64.2 and 66.9 °C. Taking into account that phosphotarget binding increases thermal stability of 14-3-3^44^, we assume that the first peak corresponds to the phosphopeptide-bound 14-3-3 core, whereas the second, poorly separated peak most likely corresponds to the ACD dimer stabilised by 1.1 °C due to the covalent attachment to the 14-3-3 core and possible intramolecular interactions. Therefore, the DSC data indicate that the ACD dimer is folded within the chimera and that the phosphopeptide binding stabilises the 14-3-3 core.

**Figure 4:**
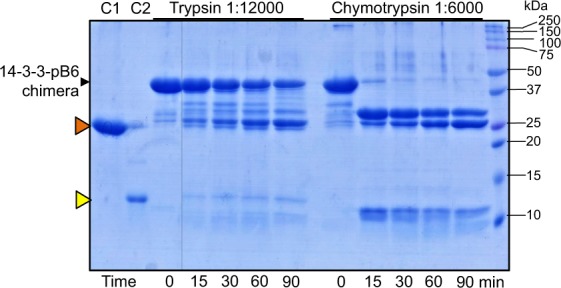
Kinetics of the 14-3-3-pB6 chimera cleavage by trypsin or chymotrypsin at indicated protease:substrate ratios. Time points are indicated below the gel in min at 37 °C. Positions of the intact chimera and protein markers bands (in kDa) are shown by arrows. C1 and C2 represent the 14-3-3σ∆C (26.1 kDa, orange triangle) and HSPB6∆N56 (11.0 kDa, yellow triangle) control lanes.

Limited trypsinolysis of the 14-3-3-pB6 chimera led to a slow disappearance of the initial band with the concomitant accumulation of two bands with apparent *M*_W_ values consistent with those of 14-3-3σ∆C and HSPB6∆N56 (~26 and ~11 kDa, respectively) (Fig. 4). In agreement with the presence of several Arg in the NTD of HSPB6 between the 14-3-3 core and ACD (e.g., Arg27, Arg32, Arg56), which are targets for trypsinolysis^33,48^, a series of fragments between 42 and 26.5 kDa could also be detected (Fig. 4). The 26.5 kDa band remains resistant to further trypsinolysis, in line with the known stability of 14-3-3 dimers.

The main site for chymotrypsinolysis in HSPB6 resides at the Tyr53-Tyr54 dipeptide in the disordered NTD, with the ACD featuring an immunoglobulin-like fold being more resistant to cleavage^48^. Incubation of the 14-3-3-pB6 chimera with chymotrypsin resulted in the accumulation of the ~11-12 kDa peptides matching HSPB6_54–153_ (10.8 kDa) and HSPB6_33-153_ (12.8 kDa) fragments. The remainder of the chimera (~42 kDa) accumulated on the gel as ~30 kDa and ~26 kDa chymotryptic products exceeding and matching 14-3-3σ∆C control (~26 kDa), respectively (Fig. 4). This is in line with the cleavage at either Phe33 or Tyr53/54 in the NTD of HSPB6. Thus, the relative resistance of the 14-3-3 and ACD parts to trypsinolysis and chymotrypsinolysis further confirmed the foldedness of these domains within the chimera.

### Isolated 14-3-3 and ACD domains show lack of direct interaction

**Figure 5:**
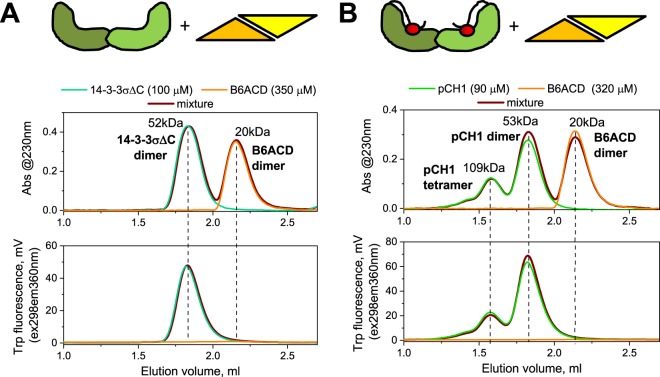
Lack of interaction between the isolated ACD and either 14-3-3σ∆C (**A**) or the 14-3-3-phosphopeptide chimera pCH1. (**B**) These two scenarios, shown schematically on top of the panels, were examined by size-exclusion chromatography with simultaneous detection by 230 nm absorbance and Trp-specific fluorescence (excitation 298 nm, emission 360 nm). Concentrations of 14-3-3 and ACD are indicated in µM. Dashed lines show the positions of the main peaks on different profiles for clarity.

**Figure 6:**
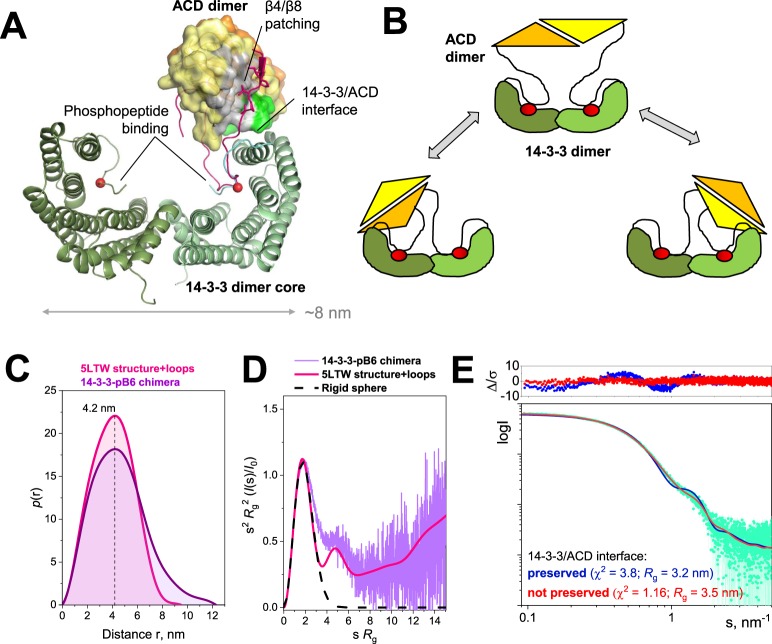
Structural analysis of the 14-3-3-pB6 chimera using SAXS. (**A**) Crystal structure of the 14-3-3σ∆C–pHSPB6∆C complex (PDB 5LTW). The 14-3-3 dimer is shown as cartoon. The HSPB6 ACD dimer is shown as a molecular surface, with residues involved in the interface with 14-3-3 highlighted in light green. The partially ordered NTD of one HSPB6 chain is shown in magenta. In addition, the N-terminal arms of the peptidic pCH1 chimera (when superimposed on the full complex) are drawn as cyan and green lines. Phosphoserines are represented by red spheres. (**B**) Schematics showing that in the absence of the β4/β8 patching, the 14-3-3/ACD interface may be preserved or not preserved. (**C**,**D**) Comparison of the *p*(r) functions (**C**) and the dimensionless Kratky plots (**D**) for the chimera (experimental SAXS data processed by *GNOM*^63^) and the 5LTW structure supplemented with the missing loops^33^ (calculated from the model by *CRYSOL*^50^ and *GNOM*^63^). (**E**) The fits of the best among each of the two types of models depending on whether the 14-3-3/ACD is preserved or not preserved to the SAXS data and the associated residuals (∆/σ) shown on top.

Given the foldedness of the 14-3-3 and ACD domains within the chimera, we questioned whether they can directly interact with each other as individual entities. Purified ACD dimer (residues 72–149) was used to probe the interaction with 14-3-3σ∆C or its chimera with the HSPB6 phosphopeptide (pCH1). We took advantage of the absence of tryptophan residues in ACD and their presence in the 14-3-3 core and employed fluorescence-assisted size-exclusion spectrochromatography (Fig. 5). As expected, 14-3-3σ∆C^44^ and ACD^47^ eluted as dimers with the apparent *M*_w_ of 52 and 20 kDa, respectively, whereas the chimera pCH1 displayed dimers (~53 kDa) and also tetramers (~109 kDa) formed due to the interdimer phosphopeptide swap^44^. However, we failed to detect any direct binding even upon loading very high micromolar concentrations of species on the column. This suggested that this secondary interface is extremely unstable on its own, but may be formed in the native complex due to the multipoint stabilising contacts observed in the crystal structure (Fig. 6A). It was most intriguing whether tethering of 14-3-3 and ACD within the chimera would permit their interaction (Fig. 6B).

### Structural analysis of the 14-3-3-pB6 chimera

We have studied the structural properties of the chimera using SEC-SAXS (Fig. 6). The SAXS profile showed linearity of the Guinier region, indicating the absence of particle interactions (aggregation or repulsion). *M*_w_ estimates from the SAXS data were in excellent agreement with the MALLS-derived value (Table 1). Pairwise distance distribution function calculated from the SAXS profile of the chimera showed a curve similar in shape to that calculated from the full-atom model of the 14-3-3σ∆C–pHSPB6∆C 2:2 complex^33^ (Fig. 6A,C); however, in case of the chimera, the right part of the plot extended further along the X-axis, resulting in the higher *D*_max_ (12.5 nm compared to 10 nm in case of the complex) and *R*_g_ value (3.51 ± 0.01 nm for the chimera vs. 3.13 ± 0.02 nm for the complex). The dimensionless Kratky plot showed the main bell-shaped peak typical of the globular proteins, a shoulder indicating the presence of another folded part/domain, and a gradual rise along the X-axis indicating the presence of flexible regions (Fig. 6D). We assume that the main peak corresponds to the chimera core based on the 14-3-3 dimer, whereas the distinct shoulder at s*R*_g_ ~ 5 is derived from ACD, which is folded within the chimera according to the DSC, SEC, and limited proteolysis data.

**Table 1:** Structural parameters of the 14-3-3-pB6 chimera determined in the SEC-SAXS experiment.

	14-3-3-pB6 chimera
**Guinier analysis**	
*I*(0) (cm^−1^)	652.5 ± 1.0
*R*_g_ (nm)	3.51 ± 0.01
s*R*_g_ range	0.34 < s*R*_g_ < 1.30
**p(r) analysis**	
*I*(0) (cm^−1^)	654.9 ± 1.0
*R*_g_ (nm)	3.57 ± 0.02
*D*_max_ (nm)	12.5
s range (nm^−1^)	0.0969–2.2797
CorMap P-value reciprocal space fit (GNOM estimate)	0.32
**Volume**, **shape and molecular weight (M**_**W**_**) analysis**	
Porod volume, nm^3^	141.4
*M*_W_ calculated from amino acid sequence, kDa	41.7•2 = 83.4
*M*_W_ from SEC-MALLS and RI concentration, kDa (*M*_W_ ratio)	82.8 (0.99)
*M*_W_ from Porod volume, kDa (*M*_W_ ratio)	88.1 (1.06)
*M*_W_ from SAXSMOW, kDa (*M*_W_ ratio)	76.9 (0.92)
*M*_W_ from *V*c, kDa, (*M*_W_ ratio)	84.2 (1.01)
**Hydrodynamic analysis (SEC-MALLS)**	
Hydrodynamic radius, *R*_h_ (nm)	4.18
*R*_g_ / *R*_h_ ratio (compared to sphere)	0.84 (0.78)
**CRYSOL (15 harmonics**, **101 points**, **constant enabled)**	
s range for model fitting	0.093–5
χ^2^, CorMap P-value	1.16, 0.027^*^
CORAL-derived model *R*_g_ (nm)	3.50

*CRYSOL fit to the SAXS data for the best *CORAL*-derived model with the non-fixed 14-3-3/ACD interface. Note: *GNOM*, *DATPOROD*, *DATMOW*, *DATVC*, *CORAL, CORMAP* and *CRYSOL* can be found as part of the ATSAS 2.8 software package^61^.

Next, we have attempted a SAXS-based structural modelling of the chimera. During modelling, the 14-3-3 core (pCH1) and the ACD dimer were considered as rigid bodies supplemented by the connecting flexible regions in *CORAL*^50^. Modelling based on the fixed relative position of the 14-3-3 core and the ACD dimer mimicking that of the 5LTW structure resulted in satisfactory fits (best χ^2^ = 3.8 in the full range of scattering data), although some oscillating residual differences between calculated and experimental scattering profiles were observed, and the smaller size of the models (*R*_g_ ~3.2 nm) could not describe the low-angle part of the experimental SAXS profile indicating a less compact size (*R*_g_ ~3.5 nm) (Fig. 6E). These results are in line with the differences observed in the *p*(r) plots (Fig. 6C). In contrast, relieving the 14-3-3/ACD interface (Fig. 6B) allowed us to obtain larger models with different ACD position relative to the 14-3-3 core which reconciled the discrepancy and provided excellent fits to the SAXS profile in the full range of scattering data (χ^2^ ranged from 1.16 to 1.27; Fig. 6E).

## Discussion

14-3-3 protein complexes are important regulatory nodes of PPI networks mediating multiple intracellular processes in norm and pathology. The constantly expanding interactome of 14-3-3 protein family requires adequate research efforts, preferably in the high-throughput format, and the ability to structurally characterise 14-3-3 complexes. This is necessary for unravelling the fundamental binding mechanisms and selectivity of 14-3-3 proteins and also for their use in drug discovery^13^. Yet, just a few structures of protein complexes involving 14-3-3 have been solved until today (PDB entries 1IB1, 5LTW, 5N6N, 6GN8). To aid in structural studies, Sluchanko *et al*. have recently proposed the chimeric approach and demonstrated its applicability to solve structures of 14-3-3/phosphopeptide complexes, which is scalable to a high-throughput format^44^.

Here we describe a 14-3-3 chimera with an almost complete phosphotarget, i.e. the relatively well-characterised partner HSPB6. We have shown that the chimera encompassing the 14-3-3 core and the major part of HSPB6 (residues 12–153) including phosphorylation of Ser16 can be readily produced in a soluble form in *E*.*coli* and has a controllable stoichiometry of binding partners (Fig. 1). Importantly, it is amenable for structural studies owing to the foldedness of the two interacting entities, the 14-3-3 dimer and the fused ACD dimer, with their dimeric interfaces preserved (Figs 2–4).

The 14-3-3-pB6 chimera dimer reproduces the main structural features of the previously characterised native 2:2 complex of 14-3-3 and pHSPB6, at the same time, forming a somewhat more flexible structure. Indeed, in the crystal structure of the 14-3-3/pHSPB6 complex, the direct interface formed between one of the 14-3-3 subunits and the ACD dimer measures only 400 Å^2^, although a salt bridge between Arg224^14-3-3^ and Glu86 ^ACD^ is present^33^. As we show here using SEC, this interface is rather unstable since an isolated ACD dimer does not bind to the 14-3-3 dimer (Fig. 5). This is in line with previous observations that also the full-length unphosphorylated HSPB6 (thus void of N-terminal binding in the AG) does not significantly interact with 14-3-3^35–37^. Importantly, the crystal structure of the 14-3-3/pHSPB6 complex reveals that the very N-terminus of HSPB6 (residues 2–10) containing the VPV motif^34^ patches the β4/β8 groove, while the immediately following phosphorylated motif is anchored in the AG of the 14-3-3 subunit (Fig. 6A). This arrangement restricts the position of the ACD relative to the adjacent 14-3-3 molecule. In the chimera, the C-terminus of 14-3-3 is linked to the HSPB6 sequence starting at position 12 only (Fig. 1A), making the patching of the β4/β8 groove on the ACD impossible (Fig. 6A). In line with that, our SAXS analysis suggests that, within the chimera, the relative position of the 14-3-3 dimer and the ACD dimer is variable (Fig. 6). In summary, our results reveal a complex dynamics of the 14-3-3/pHSPB6 association where hierarchy of stabilising factors are at play.

We assume that, like in the HSPB6 case, the secondary interfaces of other crystallographic 14-3-3 complexes may not be fully occupied in solution and require additional stabilising factors. However, this very feature could turn advantage in the future development of much more selective small-molecule stabilisers compared to targeting the primary, phosphopeptide binding regions characterised by the certain level of degeneracy and being rather similar in the most known 14-3-3/phosphotarget complexes.

Importantly, the chimeric approach may be applicable to many protein partners of 14-3-3 characterised by single, N-terminally located 14-3-3 binding phosphosites, whereas utilization of the known preferential heterodimerisation of certain 14-3-3 isoforms^3,4^ can expand this approach to study multiply phosphorylated and ternary complexes. Co-expression of 14-3-3/client chimeras can be achieved in the presence of the appropriate protein kinase(s)^45^. These concepts could see yet further progression by benefiting from the recent development of engineered *E*.*coli* strains and modified translational machinery components which enable the production of phosphoproteins using amber codon suppression^51,52^.

## Methods

### Cloning, protein expression and purification

Chimera containing the C-terminally truncated human 14-3-3σ (Uniprot ID P31947; residues 1-231, 14-3-3σ∆C) bearing on its N-terminus a His_6_-tag cleavable by 3 C protease and the phosphorylatable peptide of the small heat shock protein HSPB6 (residues 12–19), connected to the 14-3-3 core with the help of the GSGS linker (CH1), was described previously^44^. The 14-3-3σ sequence was modified to reduce surface entropy^53^ by introducing the ^75^EEK^77^ → AAA amino acid replacements (“clu3” mutant) and, after 3 C cleavage, contained three extra residues GPH at the N-terminus. The full protein chimera 14-3-3-B6 followed the same principles but contained residues 12–153 (out of 160) of human HSPB6 (Uniprot ID O14558), which includes both the Ser16 phosphopeptide region and the α-crystallin domain (ACD) (Fig. 1A). The HSPB6 part was PCR-amplified with the help of the forward B6chim_BglII 5′-ATATAAGATCTTTGCGCCGCGCCTCGGCC-3′ and B6chim_XhoI 5′- ATTAACTCGAGCTAGGCCTGTGCTGACGCTG-3′ reverse primers using the pET23 HSPB6 wild-type plasmid as a template^33^. The PCR-product was digested by BglII and XhoI restriction endonucleases and then cloned in-frame after the 14-3-3 sequence into the pET28-His-3C-CH1 plasmid digested with BamHI and XhoI restriction endonucleases. The resulting construct pET28_His_3C_14-3-3σ(clu3)∆C_B6.12-153 was verified by DNA sequencing in Evrogen (Moscow, Russia).

Chemically competent cells of the *Escherichia coli* BL21(DE3) strain were transformed using the pET28_His_3C_14-3-3σ(clu3)∆C_B6.12–153 (kanamycin resistance) and pACYC-PKA (chloramphenicol resistance)^45^ plasmids simultaneously or, alternatively, using only the chimera plasmid to produce unphosphorylated protein. Protein expression was induced by the addition of IPTG to a final concentration of 0.5 mM and continued for 4 h at 37 °C. The overexpressed proteins were efficiently purified by subtractive immobilized metal-affinity chromatography (IMAC) and gel-filtration as described for the peptide chimeras^44^. The chimera was fully soluble and could be concentrated to above 10 mg/ml for storage at −80 °C. Given the much higher yield of the chimera expressed in the absence of PKA, it was further phosphorylated *in vitro* essentially as described elsewhere^33,44,54^. The most optimal conditions leading to a complete shift of the chimera band on the native-PAGE^55^ included: 0.1 mg/ml chimera, 30 µM ATP, 0.01 mg/ml PKA, 4 mM MgCl_2_, 40 min at 37 °C. Protein concentration was determined spectrophotometrically using the sequence-specific extinction coefficient at 280 nm equal to 0.67.

PKA-phosphorylated wild-type HSPB6 and the 14-3-3σ(clu3)∆C construct were obtained in the previous work^33^. 14-3-3σ∆C, phosphorylated peptidic chimera CH1^44^, HSPB6 ACD (residues 72–149)^47^ and HSPB6∆N56 (residues 57–160)^34^ fragments were obtained as described in the previous works.

### SEC and SEC-MALLS

To study oligomerisation of the chimera, either unphosphorylated or PKA-phosphorylated samples were loaded on a Superdex 200 Increase 5/150 column (GE Healthcare) at a 0.45 ml/min flow rate. The column was equilibrated by a SEC buffer (20 mM Tris-HCl buffer, pH 7.6, containing 150 mM NaCl, 0.5 mM EDTA, 1 mM DTT, and 3% v/v glycerol) and calibrated by protein markers: BSA dimer (132 kDa), BSA monomer (66 kDa), ovalbumin (43 kDa), α-lactalbumin (14.5 kDa). The profiles were followed by absorbance at 280 nm.

The same setup was used to study direct binding between 14-3-3σ∆C or phosphorylated CH1 containing two Trp residues per chain and Trp-lacking HSPB6 ACD. In this case, the profiles were followed simultaneously by absorbance at 230 nm, and by Trp fluorescence (excited at 298 and recorded at 360 nm, at a low voltage) using fluorescence-assisted spectrochromatography on a Varian ProStar 335/ProStar 363 system. The experiments were performed three times with the most typical results presented.

The absolute masses of the species formed by the 14-3-3-pB6 chimera were analyzed on a Superdex 200 Increase 10/300 column (GE Healthcare) using multiparametric detection as described earlier^56^. Protein sample (75 µl; 10.14 mg/ml) was pre-incubated for 15 min at room temperature and then loaded on the column equilibrated by SEC buffer. The column was operated at 20 °C at a flow rate of 0.5 ml/min. Multi-angle laser light scattering (MALLS) was measured using a Wyatt Technologies miniDawn TREOS module coupled to an OptiLab T-Rex refractometer for protein quantitation (dn/dc = 0.185 was taken). The MALLS detector was calibrated relative to the scattering from toluene and, in combination with the refractometric signal, was used to determine the *M*_W_ distribution of species eluting from the SEC column.

### Small-angle X-ray scattering (SAXS) and modelling

The SAXS data (*I*(*s*) vs *s*, where *s* = 4πsin*θ*/λ, 2*θ* is the scattering angle and λ = 1.24 Å) for the 14-3-3-pB6 chimera were collected at 20 °C in parallel with the MALLS/RI detection in a SEC-SAXS format to ensure separation of the particles of interest from undesired oligomeric species and aggregates at the EMBL P12 beamline (PETRA III, DESY Hamburg, Germany^57^). This was achieved by the equal division of the flow between the SAXS (3600 × 1 s frames) and the MALLS/RI detection modules^58^, enabling simultaneous data collection. Data reduction, radial averaging and statistics analysis were done using the *SASFLOW* pipeline^59^, the SEC-SAXS data were processed using *CHROMIXS*^60^. *ATSAS* 2.8 package^61^ was used for data analysis and modelling. *PRIMUS*^62^ was used to perform Guinier analysis from which the radius of gyration, *R*_*g*_, and extrapolated forward scattering, *I*(0), were determined (ln*I*(*s*) versus *s*^2^ that were linear in the *sR*_*g*_ range reported in Table 1). The pairwise real-space distance distribution function, *p*(*r*), was calculated using *GNOM*^63^ that provided additional *R*_*g*_ and *I*(0) estimates and the maximum particle dimension, *D*_*max*_. The Porod volume and other structural parameters in solution are presented in Table 1.

In order to model the 14-3-3-pB6 chimera against the SAXS data, the crystal structure of the 14-3-3 chimera with the HSPB6 phosphopeptide (residues 12-19) (PDB 5OKF) was first modified in *Coot*^64^ to include the linker and some loop residues absent from the electron density map. This resulted in the full-atom model of the dimeric 14-3-3 core (GPH followed by residues 1–231 of human 14-3-3σ with clu3 mutations) tethered with the HSPB6 phosphopeptide (residues 12–20) by the GSGS linker (247 residues overall). The dimeric ACD part (residues 72–153) was based on the previous co-crystal structure with 14-3-3 (PDB 5LTW^33^). The first scenario was based on the relative orientation of the 14-3-3 core and ACD dimer as in the 5LTW structure (with the 14-3-3/ACD interface preserved and the N-terminal tails of the HSPB6 set flexible). The second scenario implied free movements of the ACD dimer tethered by the N-terminal tail of the HSPB6 subunits to the 14-3-3 core. In all cases, the program *CORAL*^50^ was used in multiple parallel runs to model the structure of the full dimeric 14-3-3-pB6 chimera (2 × 380 residues) by representing the missing parts of the N-terminal tail of HSPB6 (residues 21–71) in both chains (12.7% by mass) by a C_α_-trace to minimize the discrepancy between the experimental SAXS profile in a range of 0 < s < 0.3 Å^−1^ and that calculated from models. The obtained models were validated against the SAXS curve for the full range of scattering data using *CRYSOL*^50^. Two ways of interconnecting the phosphopeptides bound to 14-3-3 and the two ACD subunits have been attempted and yielded similarly fitting models.

The SAXS profile, the Kratky plot, and the pairwise distance distribution function for the full-atom model of the 14-3-3σ∆C–pHSPB6∆C complex based on the 5LTW crystal structure supplemented with missing loops were calculated using *CRYSOL*^50^ and *GNOM*^63^.

### DSC

14-3-3-pB6 chimera (1.1 mg/ml) or individual dimers of 14-3-3 and HSPB6 (1 mg/ml each) were dialysed overnight against a 20 mM HEPES-NaOH buffer, pH 7.5, containing 150 mM NaCl and subjected to DSC on a VP-capillary DSC (Malvern) at a heating rate of 1 °C/min. Thermograms were processed using Origin Pro 8.0 and transition temperature (*T*_m_) was determined from the maximum of the thermal transition^48^.

### Limited proteolysis

Proteolysis of the phosphorylated chimera (1 mg/ml) was performed at 37 °C in buffer P (20 mM Tris-HCl, pH 7.6, containing 150 mM NaCl, 15 mM β-mercaptoethanol) using either the TPCK-treated trypsin (mass ratio protease:substrate equal to 1:12,000) or the TLCK-treated chymotrypsin (mass ratio protease:substrate equal to 1:6,000) by incubating the mixtures for different times. The reaction was blocked by addition of the SDS-sample buffer containing PMSF up to the final concentration of 7 mM. The samples were then boiled and analyzed by SDS-PAGE on 15% polyacrylamide gels^65^. The apparent *M*_W_ values were determined using GelAnalyzer 2010a (http://www.gelanalyzer.com/index.html) and compared to those of 14-3-3σ∆C and HSPB6 ACD used as controls as well as calculated masses obtained using massXpert^66^.

## Data Availability

All data generated or analysed during this study are included in this published article.
